# Measurement Invariance of the Multidimensional Scale of Perceived Social Support Among Chinese and South Asian Ethnic Minority Adolescents in Hong Kong

**DOI:** 10.3389/fpsyg.2020.596737

**Published:** 2020-12-08

**Authors:** Cecilia M. S. Ma

**Affiliations:** HKCT Institute of Higher Education, New Territories, Hong Kong

**Keywords:** Chinese, measurement invariance, ethnic minority, social support, validation

## Abstract

Seven hundred adolescents (Chinese = 50%; South Asian ethnic minority = 50%) with mean age of 15.3 years (*SD* = 1.53). Multigroup confirmatory factor analysis was performed to assess measurement invariance of the MSPSS scale across Chinese and South Asian ethnic minority samples. Results show that the original three-factor structure of the MSPSS was supported in both samples. Measurement invariance was supported in terms of configural, metric, and partial scalar invariance. Given partial scalar invariance was achieved, the latent mean differences were compared across samples. Chinese adolescents had higher levels of all three types of social support when compared with their South Asian ethnic minority counterparts. The present study attempts to assess the measurement invariance of the MSPSS across ethnic groups in Asian society, which sheds light on future research that involves perceived social support among adolescents in Chinese culture contexts.

## Introduction

Adolescence is a period of life associated with significant challenges and changes (Lord et al., 1994). During this developmental period, individuals are vulnerable to adjustment difficulties and might suffer from psychological distress (McDevitt and Ormrod, 2013). Supportive social networks provide a sense of social integration and serve as a buffer against the deleterious effects of stress and uncertainty during this storm period (Wenz-Gross et al., 1997). In particular, they become more sensitive to joint influences of family and friends on their cognitive and psychosocial development during puberty (e.g., Ahmed et al., 2010; Oberle et al., 2011). Social support refers to the development of individuals’ social cognitions, knowledge, values and behaviors via the process of social exchange (Farmer and Farmer, 1996). Perceived social support can be defined as “an individual’s perceptions of general or specific supportive behaviors from people in their social network, which enhances their functioning or may buffer them from adverse outcomes” (Malecki and Demaray, 2003, p. 232). Studies have shown that adolescents’ perception of social support was positively related to better academic performance (Ahmed et al., 2010) and lower psychological distress (Ma, 2020). In fact, the effects of social support varied by social contexts (e.g., family, friends, and school) as shown in cross-sectional (e.g., Mrug and McCay, 2013) and longitudinal studies (e.g., Wang and Eccles, 2012).

### South Asian Ethnic Minority Adolescents in Hong Kong

Today, adolescents in Hong Kong are increasingly diverse. Excluding foreign domestic helpers, South Asian ethnic minority composed of 3.6% of the whole population (*n* = 263,593, Hong Kong Census and Statistics Development, 2017). Over the past decade, the number of South Asian ethnic minority adolescents born in Hong Kong has increased by 120% from 38,042 in 2006 to 81,964 in 2016 (Hong Kong Census and Statistics Development, 2017). Pakistanis, the largest proportion of this demographic group, make up 37.4% of total. Nepalese and Indians constitute 36.1% and 18.9% of the group, respectively.

Acculturation can be defined as a process in which individuals of one group adopts values and beliefs of the host culture (Berry et al., 1987). Acculturative stress defines as the type of stress related to individuals’ cross-cultural encounters, which might lead to psychological distress and behavioral problems (Berry et al., 1987). Acculturative stress may occur when ethnic minority adolescents struggle between the traditional “cultural” values (e.g., familialism) and Chinese majority culture (e.g., the pursuit of autonomy) when they are in Hong Kong (Ting, 2019). Low educational attainment and heavy financial burden may contribute perceived psychological distress among South Asian ethnic minority families (Hong Kong Unison, 2009). Compared to Chinese adolescents, they have fewer resources, which might pose an obstacle for them to adjust and adopt the values, beliefs and behaviors of Chinese majority group (Birman, 1994). In particular, they face challenges to adapt to the local community due to poor Chinese proficiency and low socio-economic status (Ku et al., 2006). In the field of ethnic minority research, greater perceived social support was found to be correlated with fewer psychological distress in response to the contextual stressors and adjustment (Novak and Kawachi, 2015). Therefore, understanding how South Asian ethnic minority adolescents perceive social support may be useful when practitioners design appropriate programs for them.

### Measurement of the Multidimensional Scale of Perceived Social Scale

When testing the perceived social support, the Multidimensional Scale of Perceived Social Support (MSPSS) has been widely used in diverse population (Vaingankar et al., 2012; Stewart et al., 2014) and adolescents (Ng et al., 2010; Lai and Ma, 2016). It is a 12-item self-report measurement tool to assess the perception of social support from friends, family, and significant others (Zimet et al., 1988). Originally, the MSPSS composed of 24 items with a 5-point Likert scale, ranging from 1 (strongly disagree) to 5 (strongly agree). Yet, the results of the factor analyses did not adequately address perceived social support (Zimet et al., 1988). Later, Zimet et al. (1990) reduced the scale to 12 items and changed the rating scale to a 7-point ranging from 1 (very strongly disagree) to 7 (very strongly agree) in order to increase response variability and minimize a ceiling effect. By using an exploratory factor analysis (EFA), they found the 12 items were loaded on three factors. There are two strengths of the MSPSS. First, it is relatively short and easy to administer. It only has 12 items and can be replicated easily to test for reliability. Second, the three-factor model of the MSPSS has been supported and translated into different languages, such as Arabic (Merhi and Kazarian, 2012), Chinese (Chou, 2000), Korean (Park et al., 2012), Malay (Ng et al., 2010), Spanish (Rey et al., 2015), Thai (Nahathai and Tinakon, 2012), and Turkish (Eker et al., 1995).

Despite the wide acceptance of the MSPSS, there are significant gaps regarding the psychometric properties of the scale. First, the factor structure of the MSPSS is somewhat mixed. Although the original three-factor model was supported (e.g., Clara et al., 2003; Stewart et al., 2014; Lai and Ma, 2016), researchers found that the 12 items were loaded onto two factors in Hong Kong Chinese adolescents (Chou, 2000; Tonsing et al., 2012) and merged into a single factor model in Pakistani pregnant women (Akhtar et al., 2010) and in American college students (Zhang and Norvilitis, 2002). One possible explanation for the variation in the factor structure of the MSPSS is that the majority of the studies (e.g., Edwards, 2004; Ng et al., 2010) have tested the factor structure of the MSPSS by using a data-driven method, such as EFA. This approach is typically used for data reduction rather than testing the theoretical framework of the scale (Fabrigar et al., 1999). Dambi et al. (2018) argued that socio-cultural contextual factors will affect the factorial validity of the scale, thereby, proposing that the original three-factor structure of the MSPSS should also be tested via confirmatory factor analysis (CFA). To date, only few researchers have employed CFA and found that the three-factor model performed better to the data than the two-factor model (Vaingankar et al., 2012; Stewart et al., 2014). Gorsuch (1983) suggested that a second-order factor model should be tested when the first-order factors are moderately correlated. Given that “factor analysis is at the heart of the measurement of psychological constructs” (Nunnally, 1978, p. 113), a more parsimonious method (e.g., CFA) to verify the factor structure of the MSPSS is warranted.

Another limitation in the psychometric properties of the MSPSS is in assessing the measurement invariance of the scale across ethnic groups. It is not clear whether individuals from culturally diverse backgrounds perceived the same trait. This is important “because without evidence of measurement invariance, it is difficult to determine whether cross-ethnic variations are due to error or measurement artifact rather than to other factors” (Sulik et al., 2010, p. 11). To make meaningful comparison across ethnic groups, measurement invariance of the MSPSS may be necessary.

To date, very few studies have been conducted to test measurement invariance of the MSPSS across ethnic groups in Chinese contexts. Under the influence of Confucianism, Chinese families emphasize on obedience, filial piety, and unquestioning loyalty (Shek, 2007). In contrast, South Asian ethnic minority families have a history of religious influence and a strong sense of ethnic origins (Ku et al., 2006). Given the social and cultural differences, the two ethnic groups might have different perceptions of social support. The present study has two goals. First, CFA was used to test the psychometric properties of the MSPSS among Chinese and South Asian ethnic minority students. The proposed hierarchical model hypothesized that three sources of social support were under *general* social support. Second, by using the multi-group confirmatory factor analysis, the present study tested the MSPSS factorial invariance across Chinese and South Asian ethnic minority adolescents in Hong Kong. We tested whether the MSPSS operates equivalently across both ethnic groups.

## Materials and Methods

### Participants

A total of 700 adolescents from Hong Kong secondary schools and communities participated in the study. Among the Chinese participants (*n* = 350), 54.3% were boys. The mean age was 15.8 (*SD* = 0.85, range 12–18 years). All participants were Chinese (100%) and selected from three secondary schools, which were chosen based on stratified sampling method (i.e., locations, gender composition, and academic performance). For the South Asian ethnic minority participants (*n* = 350), 46.9% were boys. The mean age was 14.8 (*SD* = 2.20, range from 11 to 18 years). They were recruited from one secondary school and three community centers. With regard to ethnic composition, 41% of the participants were Pakistani, with the rest of the sample having Nepalese (27%), and Filipino (25%) ethnic backgrounds. Table 1 shows an overview of the demographic characteristics of the valid samples. Missing data were below 6% in both samples (Chinese: *n* = 5, 0.01%; ethnic minority: *n* = 20, 5.7%). Descriptive statistics of both ethnic groups are shown in Table 2.

**TABLE 1:** Demographic information of the valid samples.

	Chinese (*n* = 350)	Ethnic minority (*n* = 350)
**Age**		
Mean (SD)	15.76 (0.85)	14.80 (2.2)
Range	12–18	11–18
Gender (N, %)		
Male	190 (54.3%)	164 (46.9%)
Female	160 (45.7%)	186 (53.1%)
**Ethnicity**		
Chinese	350 (100%)	
Pakistani		143 (40.9%)
Nepalese		95 (27.1%)
Filipino		88 (25.1%)
Indian		16 (4.6%)
Others		8 (2.3%)
**Birth place**		
Hong Kong	275 (78.6%)	170 (51.4%)
Mainland China	73 (21.0%)	
Other places		161 (48.6%)
	Missing = 2	Missing = 19
**Parent marital status**		
Married	276 (78.9%)	216 (72.2%)
Not married	74 (21.0%)	73 (27.8%)
		Missing = 51
**Received financial aids**		
Yes	21 (6%)	74 (24.6%)
No	276 (78.9%)	2 (0%)
Do not know	53 (15.1%)	224 (74.7%)
		Missing = 50

**TABLE 2:** Summary statistics of all items of the MSPSS by ethnic groups.

	Chinese			Ethnic minority		
Item	Mean (SD)	Skewness	Kurtosis	Mean (SD)	Skewness	Kurtosis
1	3.054 (0.434)	−0.238	−0.098	3.561 (0.563)	−0.1846	2.992
2	2.931 (0.614)	−0.379	−0.262	3.361 (0.640)	−1.234	1.078
3	2.917 (0.630)	−0.434	−0.175	3.385 (0.667)	−1.275	0.984
4	2.894 (0.517)	−0.255	−0.153	3.427 (0.599)	−1.331	1.306
5	2.869 (0.686)	−0.386	−0.369	3.391 (0.668)	−1.350	1.273
6	2.716 (0.656)	−0.152	−0.495	3.324 (0.727)	−1.105	0.378
7	2.974 (0.461)	−0.573	0.861	3.144 (0.762)	−0.910	0.218
8	3.304 (0.474)	−0.623	0.903	3.055 (0.811)	−0.632	−0.469
9	2.851 (0.544)	−0.357	0.005	3.325 (0.658)	−1.094	0.605
10	3.083 (0.556)	−0.714	0.617	3.259 (0.688)	−0.982	0.359
11	2.903 (0.693)	−0.440	−0.328	3.254 (0.787)	−1.095	0.427
12	3.029 (0.456)	−0.590	0.931	3.183 (0.839)	−0.891	−0.151
**Goodness of fit**						
Chi-square	121.144			148.592		
*df*	51			51		
RMSEA (90%CI)	0.063 (0.048–0.077)			0.073 (0.059–0.086)		
CFI	0.973			0.951		
TLI	0.965			0.937		
SRMR	0.043			0.043		

*MSPSS, Multidimensional Scale of Perceived Social Support; CFI, comparative fit index; TLI, Tucker-Lewis Index; RMSEA, root mean square error of approximation; SRMR, standardized root mean square residual; CI, confidence interval.*

### Procedures and Measurement

Chinese and South Asian ethnic minority participants completed the Chinese and English versions of the 12-item MSPSS, respectively. All participants and their parents gave informed consent to participate in the current study. 741 participants were initially invited to participate the study. Of these participants, 94.4% completed the questionnaires (*N* = 700). This study was approved by the Research and Ethnics Committee of the university. All respondents participated on a voluntary basis and received information about the purpose of the present study.

To assess the perceived social support, the 12-item version of Multidimensional Scale of Perceived Support Scale (MSPSS, Zimet et al., 1988) was employed. It assesses three sources of social support, including family support (four items), friend support (four items), and support from a significant other (four items). For example, “*My family really tries to help me*,” “*I can count on my friends when things go wrong*,” “*There is a special person in my life who cares about my feelings.*” Each item was measured on a 7-point Likert scale ranging from 1 (very strongly disagree) to 7 (very strongly agree). Higher mean scores on each subscale indicate higher levels of perceive social support. In the present study, the Cronbach alpha coefficients of the three subscales were above 0.70 (family, friends, and significant others, Table 3).

**TABLE 3:** Standardized factor loadings, comparison of latent mean estimates, Cronbach’s alphas and Cohen *d* for the MSPSS across groups.

	Chinese	α	Ethnic minority	α	Estimated mean difference	*z*	Cohen *d*
FA	Factor loading	0.83	Factor loading	0.80	0.471	6.982**	0.87
Item 1	0.815		0.706				
Item 4	0.856		0.706				
Item 6	0.766		0.642				
Item 9	0.574		0.750				
FR		0.88		0.85	0.293	6.724*	0.21
Item 7	0.848		0.726				
Item 8	0.854		0.742				
Item 10	0.777		0.808				
Item 12	0.742		0.791				
SO		0.91		0.84	0.437	7.035*	0.64
Item 2	0.856		0.767				
Item 3	0.892		0.748				
Item 5	0.871		0.769				
Item 11	0.771		0.729				
SSALL		0.91		0.90			
FM	0.550		0.913				
FR	0.767		0.649				
SO	0.920		0.960				

FM, perceived support from family; FR, perceived support from friends; SO, perceived support from significant others; SSALL, Total mean score of perceived social support. *p < 0.05, **p < 0.01.

In the present study, the South Asian ethnic minority adolescents, who generally spoke English as their second language (Hong Kong Census and Statistics Development, 2017), were invited to complete the English version of the MSPSS. This version has been administered in a sample of South Asian ethnic minority adolescents in Hong Kong (Tonsing et al., 2012). For the Chinese version, although the traditional Chinese version of the MSPSS is available, Dambi et al. (2018) reviewed the psychometric properties of the translated version of the MSPSS for non-English speaking populations and noted that the cross-cultural translation process and the structural validity of the MSPSS-C were questionable. Therefore, the author followed the backward translation guidelines as suggested by Brislin (1986) and invited two independent bilingual researchers to translate the MSPSS in order to ensure a rigorous translation process were performed. In the current study, the Chinese adolescents were asked to complete the Chinese-translated version of the MSPSS (MSPSS-C).

### Data Analysis

Multi-group confirmatory factor analysis (MGCFA) using the covariance matrix and mean structure was conducted to assess measurement invariance of the MSPSS scale across the two samples (Vandenberg and Lance, 2000). Given the assumption of normality was supported (i.e., the multivariate kurtosis ML < 3.0, the univariate skewness <2, and kurtosis <7) (Curran et al., 1996; Finney and DiStefano, 2006), maximum likelihood (ML) estimate via Mplus version 8.1 (Muthén and Muthén, 2007) was used for model estimation (West et al., 1995). To test different levels of invariance, a bottom-up approach was adopted (Meredith, 1993). Following the suggestion by Dambi et al. (2018), two different factor structure models of the MSPSS were tested in each sample (two-factor: Model 1_*Chinese*_ and Model 2_*ethnic minority*_; vs. three-factor: Model 3_*Chinese*_ and Model 4_*ethnic minority*_). Then, a hierarchical structure of the MSPSS was assessed via CFA among Chinese (Model 3a) and South Asian ethnic minority (Model 4a) adolescents separately before testing measurement invariance (Vandenberg and Lance, 2000). This method is recommended when the measurement invariance of a second-order factor model was tested (Byrne, 2005).

Once the baseline model was established, three levels of measurement invariance were tested, including configural, metric and scalar. First, configural invariance was examined to investigate whether the second-order factor model is equivalent across samples (Model 5). Second, metric invariance was tested to explore whether the factor loadings of the first-order (Model 6) and second-order factors (Model 7) are equivalent in both samples. Lastly, scalar invariance was assessed by imposing constraints on the intercepts of the measured variables (Model 8) and first-order factors (Model 9) in both samples.

Following suggestions by a number of researchers, several goodness of fit indices were assessed by the comparative fit index (CFI), the root mean square error of approximation (RMSEA), and the standardized root mean square residual (SRMR). In general, values of the comparative fit index (CFI) 0.90, Tucker-Lewis Index (TLI) > 0.90, SRMR < 0.08, and RMSEA < 0.08 reflect a reasonable fit of the model (Browne and Cudeck, 1989; Bentler, 1990; Steiger, 1990; Hu and Bentler, 1999). To evaluate whether the measurement invariance is hold, the chi-square difference test (Δχ^2^) is selected (Vandenberg and Lance, 2000). Given this test is sensitive to sample size, researchers recommended a value of change in CFI (ΔCFI) exceeding 0.01, together with a value change above 0.015 in RMSEA (ΔRMSEA) or a value change larger than 0.03 in SRMR (ΔSRMR) indicating the null hypothesis of invariance should be rejected (Chen, 2007). Partial measurement invariance may also be established when some items are not invariant (Byrne et al., 1989; Vandenberg and Lance, 2000). Modification indices (MIs) were evaluated to identify sources of misfit within a hypothesized model (Byrne et al., 1989) or non-invariant parameters in multi-group analyses (Chen et al., 2005).

## Results

### Single-Group CFAs

Compared to the two-factor model (i.e., Model 1 and Model 2), the original three-order factor model (i.e., Model 3 and Model 4) yielded better fit to the data in both samples (Table 4). This suggested that the hypothesized three-factor model showed a satisfactory fit to the data in both groups [Model 3 _*Chinese*_: χ^2^ = 121.144, df = 51, *p* < 0.01, RMSEA = 0.063 (0.05–0.08), CFI = 0.973, TLI = 0.965, SRMR = 0.043; Model 4_*ethnic minority*_: χ^2^ = 148.592, df = 51, *p* < 0.01, RMSEA = 0.073 (0.06–0.09), CFI = 0.951, TLI = 0.937, SRMR = 0.043]. In particular, the second-order factor model fit equally well in both samples [Model 3a _*Chinese*_: χ^2^ = 121.144, df = 51, *p* < 0.01, RMSEA = 0.063 (0.05–0.08), CFI = 0.973, TLI = 0.965, SRMR = 0.043; Model 4a_*ethnic minority*_: χ^2^ = 148.592, df = 51, *p* < 0.01, RMSEA = 0.073 (0.06–0.09), CFI = 0.951, TLI = 0.937, SRMR = 0.043]. Thus, the hierarchical factor structure serves as the baseline model for invariance analyses. Table 3 presents the factor loadings of all MSPSS items across both groups.

**TABLE 4:** Summary of goodness of fit indices for all models.

Model	Description	χ 2	df	RMSEA (90% CI)	CFI	SRMR	Model Comparison	Δχ 2	Δ df	Δ CFI	Δ SRMR	Δ RMSEA
1	2-factor model (Chinese)	521.099	53	0.159 (0.147–0.171)	0.819	0.10						
2	2-factor model (Ethnic minority)	180.817	53	0.082 (0.069–0.095)	0.936	0.046						
3	3-factor model (Chinese)	121.14	51	0.063 (0.05–0.08)	0.973	0.043						
3a	Second-order factor model (Chinese)	121.14	51	0.063 (0.05–0.08)	0.973	0.043						
4	3-factor model (Ethnic minority)	148.59	51	0.073 (0.06–0.09)	0.951	0.043						
4a	Second-order factor model (Ethnic minority)	148.59	51	0.073 (0.06–0.09)	0.951	0.043						
5	Configural invariance (baseline model)	269.74	102	0.068 (0.06–0.08)	0.963	0.043						
6	First-order factor loadings invariance	301.70	111	0.069 (0.06–0.08)	0.958	0.063	Model 5 vs. Model 6	31.96*	9	−0.005	0.002	0.001
7	First-order and second-order factor loadings invariance	318.68	113	0.071 (0.06–0.08)	0.955	0.080	Model 6 vs. Model 7	16.99*	2	−0.003	0.017*	0.002
8	First- and second-order factor loading, intercepts of measured variable invariance	338.48	122	0.071 (0.062–0.08)	0.953	0.082	Model 7 vs. Model 8	19.80*	9	0.002	0.002	0.000
9	First- and second-order factor loading, intercepts of measured variable, intercepts of first-order factor invariance	473.89	125	0.088 (0.080–0.097)	0.924	0.128	Model 8 vs. Model 9	135.40*	3	−0.003*	0.046*	0.017*
9a	Partial scalar intercept invariance	344.78	123	0.071 (0.062–0.080)	0.952	0.086	Model 8 vs. .Model 9a	6.30*	1	−0.001	0.004	0.000

CFI, comparative fit index; TLI, Tucker-Lewis Index; RMSEA, root mean square error of approximation; SRMR, standardized root mean square residual; CI, confidence interval; Δ, change in value. *p < 0.05.

### Measurement Invariance

First, the configural model was examined in Model 5. This model fit the data well [χ^2^ = 269.74, df = 102, *p* < 0.01, RMSEA = 0.069 (0.06–0.08), CFI = 0.963, TLI = 0.953, SRMR = 0.043], indicating the MSPSS measures the same constructs between two groups. As configural invariance holds, the invariance of factor loadings of first-order (Model 6) and second-order factors (Model 7) were investigated. Both models fit the data well as indicated by the non-significant Δ CFI (Δ CFI _*Model* 6 vs_._Model 7_ = 0.00), Δ RMSEA (Δ RMSEA_Model 6 vs_._Model 7_ = 0.00), Δ SRMR (_*Model* 6 vs_._Model 7_ = 0.00). This shows that all factor loadings were equivalent across samples. Therefore, the scalar invariance was examined by imposing equality constraints on item intercepts (Model 8). The hypothesis of item intercept invariance was supported as indicated by the non-significant changes in CFI (i.e., Δ CFI = 0.00), RMSEA (i.e., Δ RMSEA = 0.00), and SRMR (i.e., ΔSRMR = 0.00). In Model 9, the intercepts of all first-order factors were constrained to be equal across samples. This model did not fit the data as indicated by the significant changes in CFI (Δ CFI = 0.03), RMSEA (i.e., Δ RMSEA = 0.02), and SRMR (i.e., Δ RMSEA = 0.05). Examination of modification indices suggested that the intercepts of two first-order factors (i.e., perceived family support and perceived support from a significant other) were allowed to vary across samples. Partial measurement invariance is acceptable when the proportion of non-invariant parameters to all parameters tested was less than 20% (Dimitrov, 2010). Specifically, Rudnev et al. (2018) noted that comparison of the latent means across groups can be tested when at least two of the factor loadings and intercepts are held equal. The non-invariant parameters were freely estimated in the modified model (Model 9a). The hypothesis of partial invariance is acceptable as indicated by the insignificant changes in CFI (Δ CFI = 00), RMSEA (i.e., Δ RMSEA = 0.00), and SRMR (i.e., ΔSRMR = 0.00). This indicated that both Hong Kong Chinese and South Asian ethnic minority adolescents used same metric in responding to the items of the MSPSS.

With acceptance of the partial scalar invariance, the latent mean of the first-order factor is tested (Chen et al., 2005). To investigate group differences in latent factor mean, equality constraints were imposed on latent factor means of the South Asian ethnic minority sample while the latent factor means of the Chinese sample were freely estimated (Byrne, 2005). Chinese adolescents perceived significantly higher social support from family (estimate = 0.47, *z* = 6.98, *p* < 0.01), friends (estimate = 0.29, *z* = 6.72, *p* < 0.05), and the significant other (estimate = 0.44, *z* = 7.04, *p* < 0.05) than their ethnic minority counterparts (Table 3). By using Cohen’s *d* (Cohen, 1992), the group differences were larger in perceived family support (*d* = 0.87) and perceived significant support (*d* = 0.64) than in perceived friends support (*d* = 0.21).

## Discussion

The purpose of the present study was to test the psychometric properties of the MSPSS in terms of its factor structure and measurement invariance in both Chinese and South Asian ethnic minority adolescents. Results of the CFA provide evidence for the construct validity of the MSPSS for both Chinese and South Asian ethnic minority adolescents in Hong Kong. Unlike the past research (Chou, 2000; Tonsing et al., 2012), adolescents, regardless of ethnicity, were able to differentiate three sources of perceived social support. This result corresponds with the original three-factor model of the MSPSS, which has been found in Mexican American (Edwards, 2004), Haitian (Hannan et al., 2016), and Turkish (Eker et al., 2000) samples. Moreover, the hierarchical model of social support was shown and consistent with past research (Cheng and Chan, 2004; Vaingankar et al., 2012). All three primary factors have moderate to high loadings (Chinese:0.55 to 0.92; ethnic minority:0.65 to 96) on the general social support, which were consistent with Clara et al. (2003) study.

In particular, configural invariance results show that the first-order and second-order factor structure of the MSPSS was equivalent across ethnic groups. Results of this study suggest that the hierarchical structure of the MSPSS is considered as the same between the two groups (Vandenberg and Lance, 2000; Byrne, 2005; Chen et al., 2005). This contradicts to the notion that respondents in collectivistic contexts might not be able to differentiate the social support between family and the significant others (Akhtar et al., 2010). Indeed, the three-factor structure of the MSPSS was also found in Malaysian young adults (Guan et al., 2015). Clearly, more researches are needed to explore the factor structure of the MSPSS, especially in other contexts.

Multi-group confirmatory factor analysis provided evidence for the metric invariance of the MSPSS. This indicates that the use of the observed items to assess these underlying factors of the MSPSS is likely equivalent for both ethnic groups (Vandenberg and Lance, 2000). The present study shows that the intercepts of the first-order factors were not the same across groups. In particular, partial measurement invariance results suggested that there are differences between Chinese and South Asian ethnic minority adolescents in terms of the intercepts of the perceived support from family and significant others. It is noteworthy that the non-invariant sources of the two intercepts of the first-order factors do not affect the comparability of the MSPSS significantly across two groups (Steinmetz, 2013).

The ethnic differences in the latent means are somewhat expected and is consistent with prior research. For example, based on a sample of American college students, Osman et al. (2014) found that full scalar measurement invariance was not supported across genders. Latent mean differences in the first-order factors suggest that Chinese adolescents perceived higher social support from all sources than their South Asian ethnic minority counterparts. These findings are consistent with past research (Loper, 2004; Ma, 2020), Chinese adolescents usually have a better network than their South Asian minority counterparts. The presence of such differences sheds light on the use of the MSPSS when researchers compare the means scores of the MSPSS subscales among Chinese and South Asian ethnic minority adolescents. Researchers should be cautions when they compare the mean differences on the items or subscales of the MSPSS.

Despite evidence showing that the MSPSS is a psychometrically sound instrument, there are some limitations in the study. First, a cross-sectional design was adopted in the present study. There is a need to explore whether the construct is stable across time via longitudinal invariance tests. Second, the focus of this study was to test the measurement invariance of the MSPSS. There is a need to examine the buffering effect of the perceived social support on psychological well-being based on a prospective design. Also, strict measurement invariance is not tested in the present study. It would be insightful to explore whether the invariance of error covariance operates equivalently, which has been considered too stringent (Millsap and Kwok, 2004; Byrne, 2005) and of little practical interest (Widaman and Reise, 1997). Lastly, future investigations should replicate the findings of the present study with Chinese samples in other Asian contexts.

One of the strengths of the study is to assess the measurement invariance of the MSPSS in both Chinese and South Asian ethnic minority adolescents in a non-western context. Findings in the current study imply that the three-factor conceptual model of the MSPSS is supported in Chinese contexts. The results of the measurement invariance suggest that both ethnic groups endorsed the same response categories when they perceived support from three different social resources. It found that the MSPSS is invariant across Chinese and South Asian ethnicity minority groups, suggesting that the MSPSS can be utilized to measure the perceived social support in these two groups. Second, this study was conducted based on a relatively large sample (*N* = 700). Third, the current study employed a sophisticated method for testing psychometric properties and measurement invariance of the MSPSS across samples. The variations in the latent mean scores imply that ethnic differences should be taken into account when researchers compare perceived social support across cultures. Lastly, this is the first study to assess the measurement invariance of the MSPSS in both Hong Kong Chinese and South Asian ethnic minority adolescents in Chinese contexts.

In sum, the MSPSS operates equivalently across Chinese and South Asian ethnic minority adolescents. The findings of the present study support the three sources of perceived social support of the MSPSS. Differences found in the study can shed light on how to design appropriate interventions for adolescents from diverse backgrounds. This study extends our understanding of the use of the MSPSS among adolescents in Asian contexts.

## Data Availability Statement

The raw data supporting the conclusions of this article will be made available by the authors, without undue reservation.

## Ethics Statement

The studies involving human participants were reviewed and approved by The Hong Kong Polytechnic University. Written informed consent to participate in this study was provided by the participants’ legal guardian/next of kin.

## Author Contributions

CM conducted the study, collected the data, performed the data analysis, and drafted the manuscript.

## Conflict of Interest

The author declares that the research was conducted in the absence of any commercial or financial relationships that could be construed as a potential conflict of interest.
